# Deep-UV excitation fluorescence microscopy for detection of lymph node metastasis using deep neural network

**DOI:** 10.1038/s41598-019-53405-w

**Published:** 2019-11-15

**Authors:** Tatsuya Matsumoto, Hirohiko Niioka, Yasuaki Kumamoto, Junya Sato, Osamu Inamori, Ryuta Nakao, Yoshinori Harada, Eiichi Konishi, Eigo Otsuji, Hideo Tanaka, Jun Miyake, Tetsuro Takamatsu

**Affiliations:** 1 Department of Pathology and Cell Regulation, Kyoto Prefectural University of Medicine, 465 Kajiicho, Kawaramachi-Hirokoji, Kamigyo-ku, Kyoto 6028566 Japan; 2 Division of Digestive Surgery, Department of Surgery, Kyoto Prefectural University of Medicine, 465 Kajiicho, Kawaramachi-Hirokoji, Kamigyo-ku, Kyoto 6028566 Japan; 3 Institute for Datability Science, Osaka University, 2-8 Yamadaoka, Suita, Osaka 5650871 Japan; 4 Faculty of Medicine, Osaka University, 2-2 Yamadaoka, Suita, Osaka 5650871 Japan; 5 Department of Surgical Pathology, Kyoto Prefectural University of Medicine, 465 Kajiicho, Kawaramachi-Hirokoji, Kamigyo-ku, Kyoto 6028566 Japan; 6 Global Center for Medical Engineering and Informatics, Osaka University, 1-3 Yamadaoka, Suita, Osaka 5650871 Japan; 7 Department of Medical Photonics, Kyoto Prefectural University of Medicine, 465 Kajiicho, Kawaramachi-Hirokoji, Kamigyo-ku, Kyoto 6028566 Japan

**Keywords:** Cancer imaging, Metastasis, Wide-field fluorescence microscopy, Biomedical engineering, Machine learning

## Abstract

Deep-UV (DUV) excitation fluorescence microscopy has potential to provide rapid diagnosis with simple technique comparing to conventional histopathology based on hematoxylin and eosin (H&E) staining. We established a fluorescent staining protocol for DUV excitation fluorescence imaging that has enabled clear discrimination of nucleoplasm, nucleolus, and cytoplasm. Fluorescence images of metastasis-positive/-negative lymph nodes of gastric cancer patients were used for patch-based training with a deep neural network (DNN) based on Inception-v3 architecture. The performance on small patches of the fluorescence images was comparable with that of H&E images. Gradient-weighted class activation mapping analysis revealed the areas where the trained model identified metastatic lesions in the images containing cancer cells. We extended the method to large-size image analysis enabling accurate detection of metastatic lesions. We discuss usefulness of DUV excitation fluorescence imaging with the aid of DNN analysis, which is promising for assisting pathologists in assessment of lymph node metastasis.

## Introduction

The number of cancer patients has been increasing with the worldwide growth and aging of the population in these past decades. Thanks to the advances in diagnostic and therapeutic modalities, the prognosis for cancer patients has improved. Nevertheless, lymph node (LN) metastasis is still one of the most important prognostic factors in some common malignancies (e.g. gastric cancer, breast cancer). To provide cancer patients with appropriate treatments, accurate evaluation for the presence of LN metastasis is necessary^1,2^. Histopathological analysis allows accurate and convincing detection of LN metastasis.

Formalin fixed, paraffin embedded (FFPE) tissue specimen is used for gold-standard histopathology. It requires multiple steps including fixation, dehydration, paraffin embedding, deparaffinization, staining, and more for preparation. These steps take a few days or longer, and hence are not implementable in rapid diagnosis during surgery. Alternatively, frozen section analysis is widely used for intraoperative diagnosis. The excised specimens are rapidly frozen, thin-sliced, stained with hematoxylin and eosin (H&E) and diagnosed by a pathologist. These procedures generally require about 30 minutes in total. However, there are some problems in this process. First, it is relatively time-consuming, considering the situation under surgery. Second, preparation of the frozen specimen requires experienced skills, particularly in the steps of rapid freezing and thin-slicing. Moreover, the formation of ice crystals due to rapid freezing often leads to degradation of sample quality^3^. Thus, histopathological examination of LN metastasis during surgery requires a diagnosis support.

To address the current issue in the intraoperative diagnosis, we focus on deep-UV (DUV) excitation fluorescence imaging. Deep-UV excitation effectively limits the excitation volume to a thin layer near the surface of the tissue due to the narrow depth of penetration^4^. Using this property, MUSE (microscopy with ultraviolet surface excitation) technique has been reported^5–9^, which removes the requirement for physical thin-sectioning of the tissue and thus speeds up the overall microscopy process by eliminating nearly all of the preparation steps required for conventional histopathology. Regarding to the intraoperative diagnosis procedure, the MUSE technique can omit the steps of rapid freezing and thin-slicing. In addition, the process of fluorescence staining for MUSE is performed in a shorter time compared to H&E staining, which can also contribute to rapid diagnosis. Recently, we established the fluorescent staining protocol for DUV excitation fluorescence imaging^9^ that has enabled clear discrimination of nucleoplasm, nucleolus, and cytoplasm, which are important organelles in pathological diagnosis.

Although DUV excitation fluorescence images are good enough for pathologists to identify metastatic lesions in LNs, manual inspection of the fluorescence images by pathologists is not practical; pathologists should be trained to examine the fluorescence images. Even with the conventional H&E slides, assessment of LN metastasis is laborious for pathologist. Additionally, a retrospective study reported that nodal statuses identified by skilled and inexperienced pathologists were inconsistent in 24% of patients^10^.

These issues arising from the manual assessment of LN metastasis can be overcome with a machine learning approach. Thanks to advancement of such approaches in these past few years, deep neural network (DNN), demonstrating outstanding performances in automated image-recognition applications^11–15^ including accurate detection of metastatic lesions in H&E images of LNs^16–19^, is expected to assist pathologists in identifying metastasis as rapidly and accurately as possible. The aim of this study is to examine accuracy, validity and usefulness of DUV excitation fluorescence imaging with the aid of DNN analysis for detection of LN metastasis in gastric cancer patients. Results are compared with those obtained by DNN analysis of H&E images.

## Results

### Fluorescence imaging and histological inspection of LNs of gastric cancer patients

**Figure 1:**
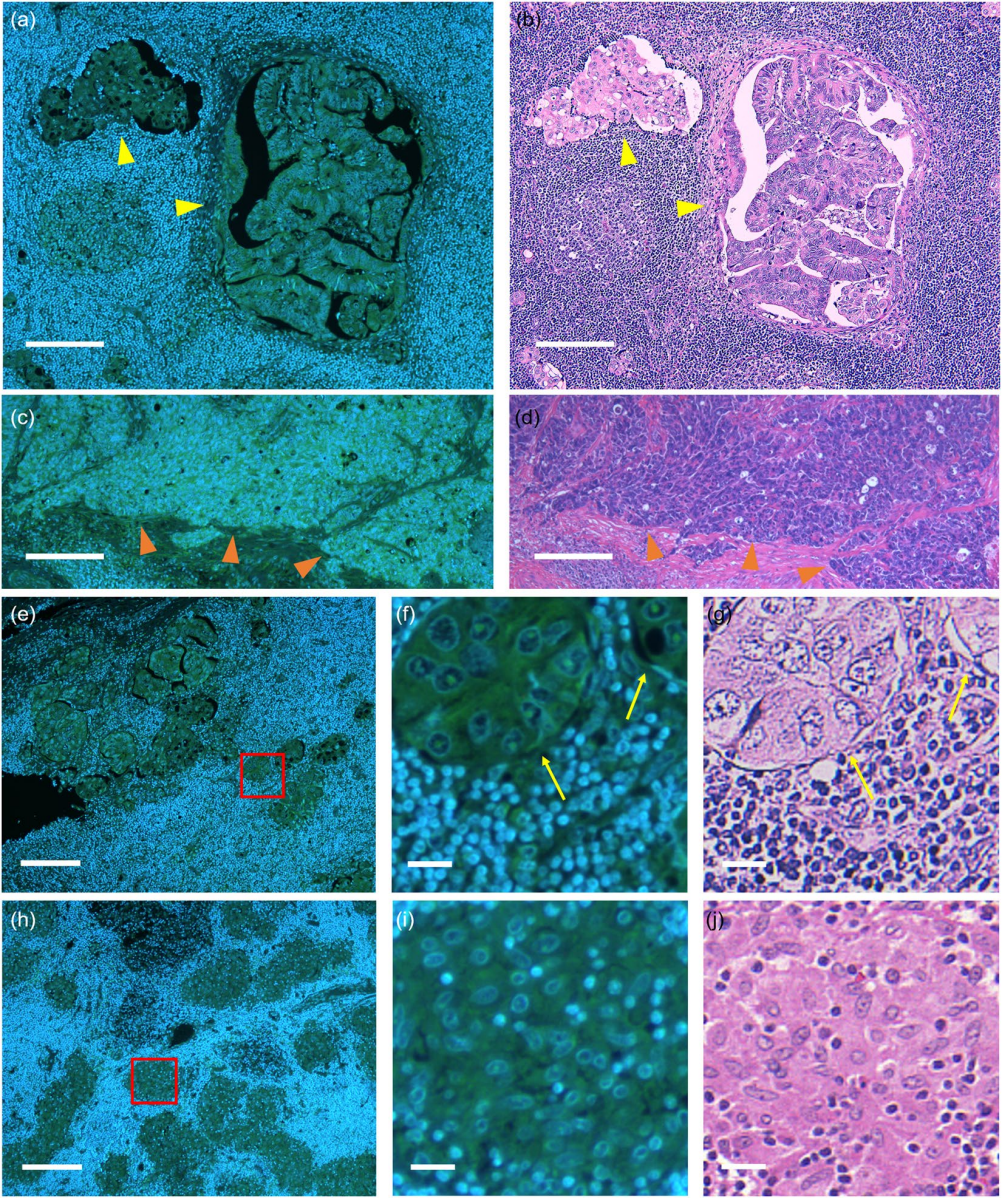
Fluorescence (**a**,**c**,**e**,**f**,**h**,**i**) and H&E (**b**,**d**,**g**,**j**) images of thin-sliced FFPE LNs of gastric cancer patients. Mixed types of glandular and cribriform patterns are exhibited (yellow arrowheads) in (**a**,**b**), while a solid tumor pattern is exhibited (orange arrowheads) in (**c**). Digitally magnified images displaying cancer cells and histiocytes are shown in (**f**,**g**) and (**i**,**j**), respectively. Yellow arrows indicate metastatic lesions. (**f**,**i**) are magnified images of the section marked by red boxes in (**e**) and (**h**), respectively. Nucleus: blue, cytoplasm and nucleolus: green (**a**,**c**,**e**,**f**,**h**,**i**). All the images were acquired with an objective lens of 10 × magnification. Scale bars show 200 μm (**a–d**,**e**,**h**) and 20 μm (**f**,**g**,**i**,**j**).

We acquired fluorescence images of thin-sliced, FFPE LNs of gastric cancer patients with the DNA and RNA labeling protocol. Representative images are shown in Fig. 1, together with H&E images of corresponding serial sections. Glandular tissue and cell structures such as nuclei, nucleoli, and cytoplasm appear in the fluorescence images as vividly as in the H&E images, without requiring time-consuming processes. In the H&E images, the cancer cells metastasized were identified by a pathologist. Cells with similar structural characteristics were identified in the fluorescence images.

The metastatic lesions seen in Fig. 1 exhibit diverse characteristic structures. Those can be classified by histologic type, i.e., well-differentiated, moderately-differentiated, and poorly-differentiated adenocarcinoma. Figures 1a,b present a mixed type of well- and moderately-differentiated adenocarcinoma, in which cancer cells form glandular and cribriform pattern respectively, while Fig. 1c,d present a poorly-differentiated type, which forms solid pattern. The histological differences in these cells are recognized well in both the fluorescence and H&E images whereas the boundaries between cancer cells and normal LN tissues are clearer in the fluorescence images than the H&E images.

Representative images of metastatic and non-metastatic lesions are shown in Fig. 1e,h and magnified views of a metastatic lesion in Fig. 1f,g. Lymphocytes and cancer cells are identified well; lymphocytes appear as blue round structures represented by nuclear DNA stained with DAPI as they have few cytoplasmic components, whereas cancer cells have abundant cytoplasmic components. Collection of inflammatory cells (e.g., histiocytes) can look similar to the lesion at a glance (Fig. 1h,i,j), but by glancing at the intracellular structures we can discriminate the cancer cells from the histiocytes; compared to histiocytes, cancer cells show distinct features, such as a higher nuclear-to-cytoplasmic ratio, nucleomegaly, and larger and clearer nucleoli. Hence, the morphological information gained through the DNA and RNA fluorescence images is useful for histological inspection of LN metastasis.

### Data set and DNN model for detection of LN metastasis (DNN analysis of the fluorescence images for detection of LN metastasis)

**Figure 2:**
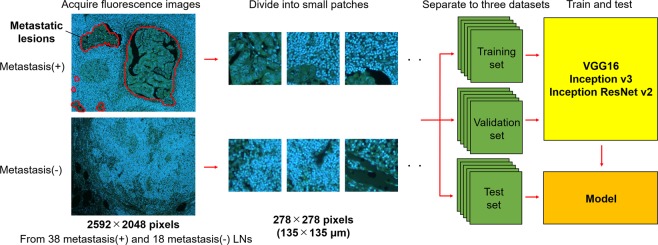
Workflow of establishing DNN models. From the fluorescence images of metastasis-positive LNs, only the small patches including metastatic lesions (marked by red lines) are used. Fluorescence images (2,592 × 2,048 pixels) were acquired at 10 × magnification of objective lens from 38 metastasis-positive and 18 metastasis-negative LNs. Each image was divided into nonoverlapping patches with 278 × 278 pixels. The patches were classified by the authors into positive and negative. Only the patches containing metastatic lesions were used as positive data. The negative patches were used only from non-metastatic LNs. We prepared a total of 27,712 fluorescence patches, then divided them into training, validation, and test sets, respectively. Three DNN architectures were trained and tested.

We used the DNA and RNA fluorescence images for DNN analysis of LN metastasis (Fig. 2). The entire workflow of our analysis is summarized in Suppl. Figure 1. For the DNN analysis, we first collected a large number of fluorescence images of metastatic and non-metastatic LNs of gastric cancer patients. Detailed protocols and policies for the data collection are described in Materials and Methods. After the data collection, individual images were divided into small patches so that a Graphics Processing Unit (GPU) could perform DNN analysis. The small patches were classified by pathologists into metastatic and non-metastatic groups and further subgrouped into training, validation, and test datasets, with consideration that the patches obtained from one LN were not included in more than one dataset. The training and validation datasets were used for developing a DNN model. We used each of three open source algorithms including VGG16^20^, Inception v3^21^, and Inception ResNet v2^22^ for developing the DNN model. Each DNN model classified the test datasets. Similarly, we used H&E images to develop and examine DNN models. The number of patches included in each dataset is shown in Suppl. Table 1.

The classification accuracies for fluorescence and H&E images analyzed with the individual algorithms are shown in Table 1. The values in each row shows the results of ten times analyses where initial values of the weights selected from uniform distribution from −0.05 to 0.05 in the network were varied, meaning that the influence of the initial values was somewhat calibrated in the values shown in the table. The mean values of the accuracy in fluorescence patch classification with the three algorithms were 97.4%, 98.2%, and 97.9%, respectively. These values were comparable to the results of H&E patch classification. Additionally, the maximal accuracies were also similar between the fluorescence (98.8%) and H&E (99.4%) classifications. Area under the curve (AUC) in the ROC (receiver operating characteristic) curves for the results with the highest accuracies (Suppl. Figure 2) was 0.999 for both the fluorescence and H&E cases. Hence, the DNN analysis with the fluorescence images allows detection of LN metastasis as highly accurate as that with the H&E images.

**Table 1:** Accuracies of individual algorithms in classifying the small patches of metastasis-positive and metastasis-negative LNs.

Model	Image type	Mean (%)	Maximum (%)	SD (%)
VGG16	Fluorescence	97.4	98.7	0.946
VGG16	H&E	97.6	98.2	0.325
Inception v3	Fluorescence	98.2	98.8	0.333
Inception v3	H&E	98.7	99.4	0.654
Inception ResNet v2	Fluorescence	97.9	98.6	0.461
Inception ResNet v2	H&E	97.0	98.8	1.53

SD = Standard deviation.

We further analyzed the Inception v3 model with the weights that attained the highest accuracy in the ten times analyses. The DNN structure based on Inception v3 is shown in Suppl. Figure 3. The model classified 1,843 patches into positive (including cancer cells) and 1,987 patches into negative (not including cancer cells). Although small in number, false predictions were included in the results (28 false positive and 19 false negative). The representative images of individual error cases are shown in Suppl. Figure 4. In the false-negative case, lymphocytes cover the most area, while a few cancer cells appear only at a corner. On the other hand, the false-positive image includes connective tissue, which can be regarded as a part of the glandular structure formed by cancer cells.

**Figure 3:**
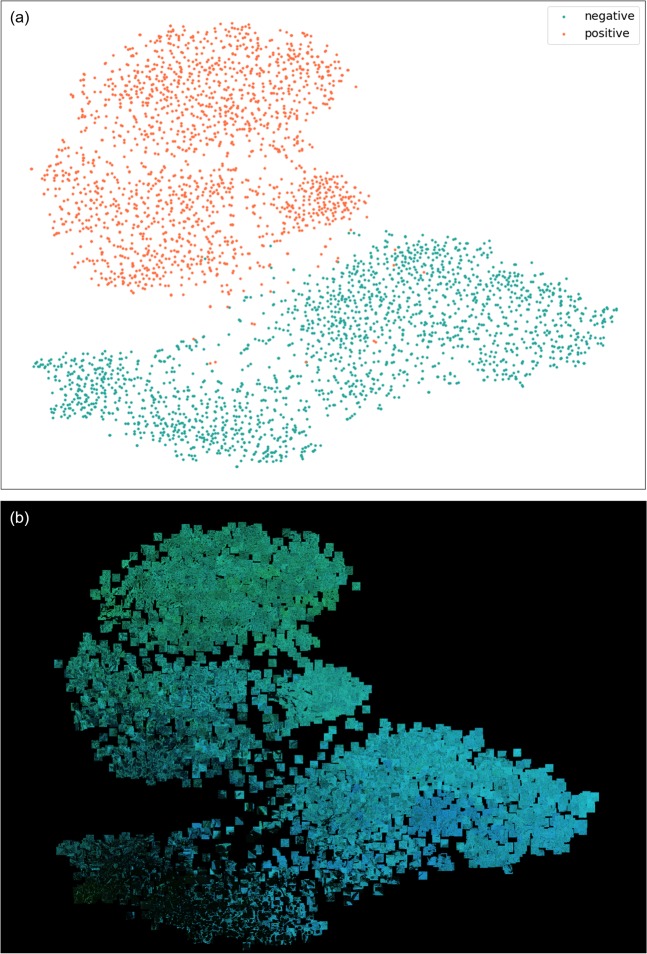
Illustration of the metastasis classification results in a two-dimensional space via the t-SNE algorithm. (**a**) Shows how the algorithm classified the patches. The orange and green plots indicate the prediction of metastasis-positive and -negative, respectively. (**b**) Shows how the classified images were distributed in the t-SNE illustration. N = 3,828 images (test data) of 38 metastasis-positive and 18 metastasis-negative LNs from 20 patients are shown in each view.

We also analyzed the internal features revealed by the DNN, using t-distributed stochastic neighbor embedding (t-SNE)^23^ (Fig. 3). The 2,048-dimensional input value (the number of dimensions after global average pooling) to the layer immediately before the final layer was compressed into two dimensions by t-SNE. A total of 3,828 test data images were compressed and plotted in two-dimensional space. Metastasis-positive and metastasis-negative patches were clearly divided into two clusters.

**Figure 4:**
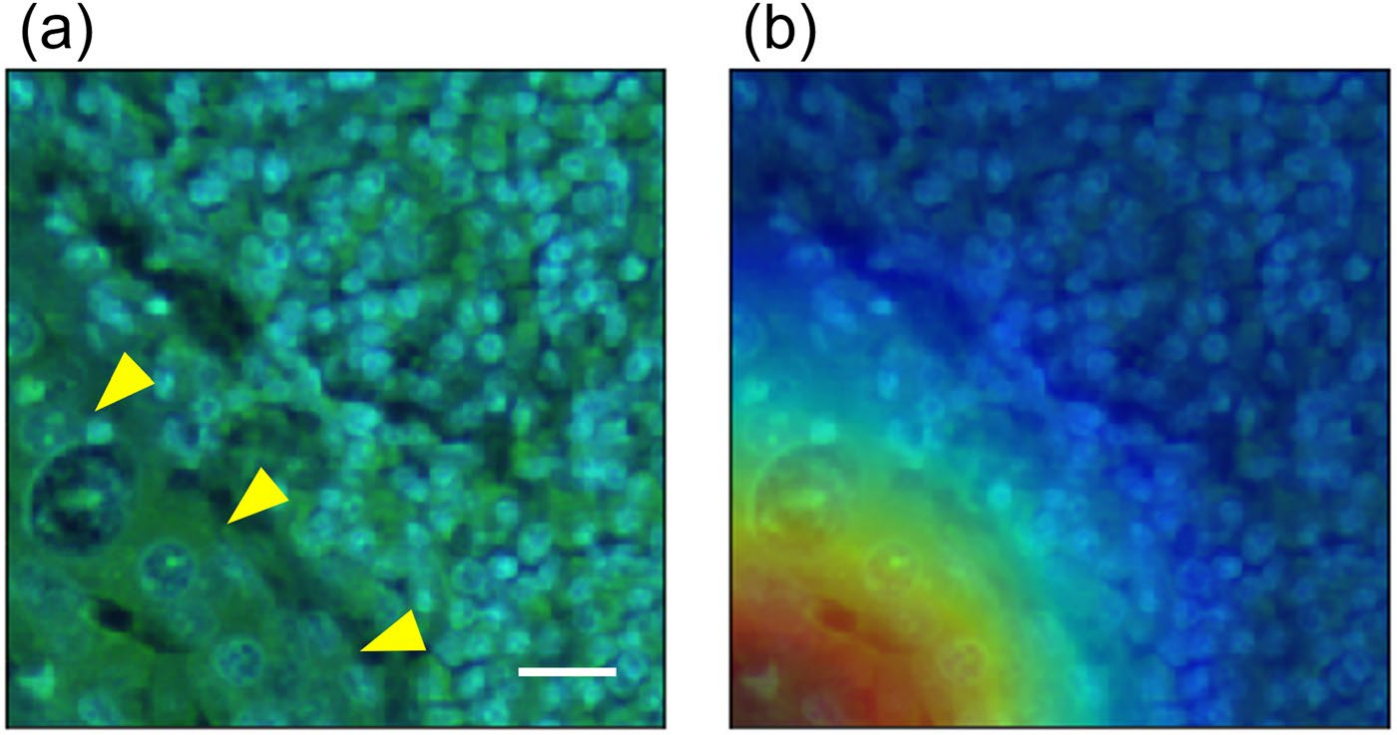
Representative result of Grad-CAM analysis for true positive prediction. (**a**) A raw fluorescence patch containing a metastatic lesion. Yellow arrowheads indicate the metastatic lesion. (**b**) The class activation map (heatmap) showing the region of the patch that had greatest weight in the DNN classification. Scale bar, 20 μm.

To reveal how the DNN algorithm classified the metastasis-containing patches, we performed gradient-weighted class activation mapping (Grad-CAM)^24^, which can highlight the area where DNN focused upon the classification with a heat map. The Representative mapping result is shown in Fig. 4. This result indicated that DNN focused on the red area upon the classification. The red area is roughly consistent with the metastatic lesion.

### Sliding-window image processing for large-size images

**Figure 5:**
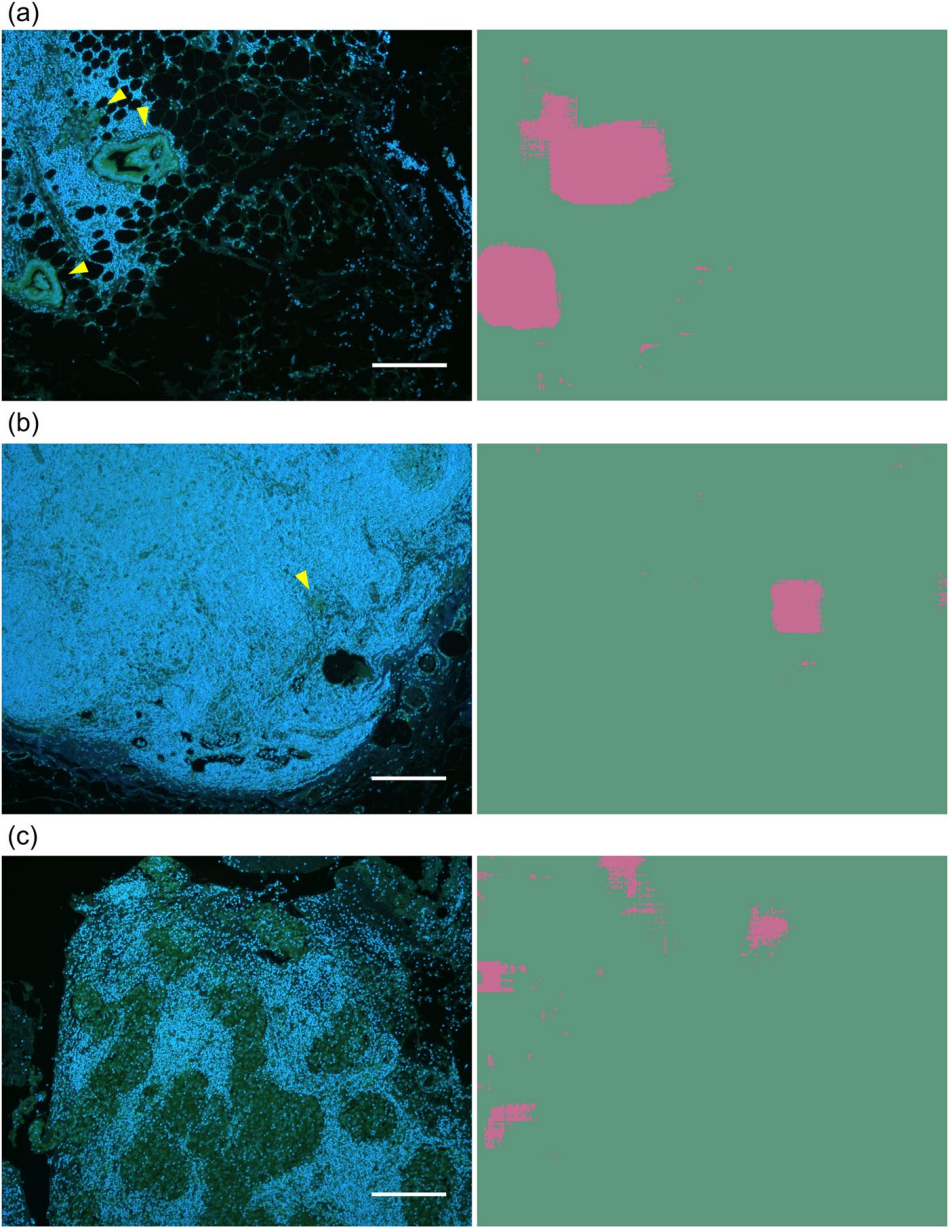
Representative results of the sliding-window image processing applied to the large-size images and their corresponding fluorescence images. The pink and green areas in the right images indicate the prediction pixels of metastasis-positive and -negative areas, respectively. (**a**,**b**) Metastasis-positive cases. Yellow arrowheads indicate metastatic lesions. The positive prediction areas are slightly larger than the actual cancer area since the output value was assigned to the pixel corresponding to the center of the 278 × 278 pixels window. Isolated metastatic lesion is detected in (**b**). (**c)** A metastasis-negative case. Nodules, consisting of histiocytes, are misclassified as metastatic lesions. Scale bars: 200 μm.

The goal of this study is not the small patch analysis but a whole slide (containing a whole LN) image analysis. To approach the goal, we investigated whether the patch analysis could be extended to undivided images consisting of a larger number of pixels (2,592 × 2,048). Another 100 fluorescence images to conduct this investigation were acquired from metastatic and non-metastatic LNs which were not employed for development and evaluation of the DNN models. For each of the new images, we applied the following method: the image was extended to a 2,870 × 2,325 pixels image by the mirroring process (see Methods); a 278 × 278 pixels region at the corner was analyzed by the DNN model to obtain the output value as the positive prediction probability; the output value was rounded off to be 1 (positive) or 0 (negative), and subsequently assigned to the pixel corresponding to the center of the 278 × 278 pixels image; these procedures were repeated with sliding of the analysis window by 10 pixels in X or Y directions until the whole area was analyzed. Representative resultant images and corresponding fluorescence images are shown in Fig. 5. Figure 5a,b show the results for metastasis-positive cases with glandular structures formed by metastatic cancer cells (Fig. 5a) and a small cluster of metastatic tumor cells (Fig. 5b).

The lesions where these structures are seen in the fluorescence images match approximately with the positive prediction region in the analyzed image, although the positive prediction area is slightly larger than the actual cancer area. It is particularly noticeable that the DNN model could detect small lesions (54.6 × 42.8 μm in size) (see Fig. 5b**)**. In fact, all the analyzed 100 metastasis-positive images contain positive prediction points and hence can be diagnosed as metastasis-positive cases.

On the other hand, some regions where cancer cells are not present are misclassified in Fig. 5a,b. More obviously, many nodules which consist of activated histiocytes, so-called epithelioid cells, are misclassified as metastatic lesions in the metastasis-negative case (Fig. 5c). In fact, 99 of the analyzed 100 metastasis-negative images contained at least one pixel diagnosed as positive.

There can be at least one reason for such high rate of misclassification for the large-size images. Each of the large-size images consists of 53,300 prediction results of the small patches. Simply considering the classification accuracy of the DNN model based on Inception v3, i.e., 98.8%, we can estimate that 640 misclassification points can be included in each image. This value can be overestimated because a region that is likely to be mistaken for metastasis-positive such as inflammation can contain the misclassified points with higher probability, but 53,300 predictions can contain a misclassification from the probabilistic point of view.

### Majority decision processing and large-size image analysis

To improve the accuracy for the large-size images, we introduced a majority decision rule (see Methods for details). This idea is based on the fact that a specific metastatic cancer area is included in the multiple images cropped with the sliding window and each of the multiple images is classified as either metastatic-positive or metastatic-negative. In such a case, if we set certain threshold at proportion of the number of images classified as metastatic-positive to the number of the multiple images, the specific region can be classified as metastatic-positive while the probabilistic misclassification can be compensated. Therefore, the majority decision model can theoretically detect small tumors of 0.5 µm in size considering the resolution of the window. With this approach, the prediction accuracy increased for the large-sized images, as shown in Table 2. By setting the threshold at 70%, the highest accuracies of true positive and negative predictions, at 96%, were attained.

**Table 2:** True prediction rates for large-size images processed with and without the majority decision model having various thresholds.

	Threshold	True prediction rates	
		Metastasis-positive	Metastasis-negative
Without majority decision processing		100/100	1/100
With majority decision processing	40%	99/100	77/100
	50%	98/100	86/100
	60%	97/100	93/100
	70%	96/100	96/100
	80%	94/100	97/100
	90%	92/100	99/100

In the process of mapping the metastatic cancer area and performing majority decision processing to determine whether 200 images are positive or negative, the time taken for the entire process mainly depends on the sliding-window step size at mapping. When the step size is 10 pixels, it takes 840 sec to map one image. Increase in step size to 35 and 70 pixels can reduce the process time to 69 and 17 sec, respectively. Diagnostic accuracies for these step sizes are shown in Suppl. Table 2. At the threshold of 70%, the accuracies were hardly decreased by the increases in step size. It is further worth noting that processing time can be reduced when multiple GPUs are used for parallel processing or when a GPU with a higher processing speed is used.

**Figure 6:**
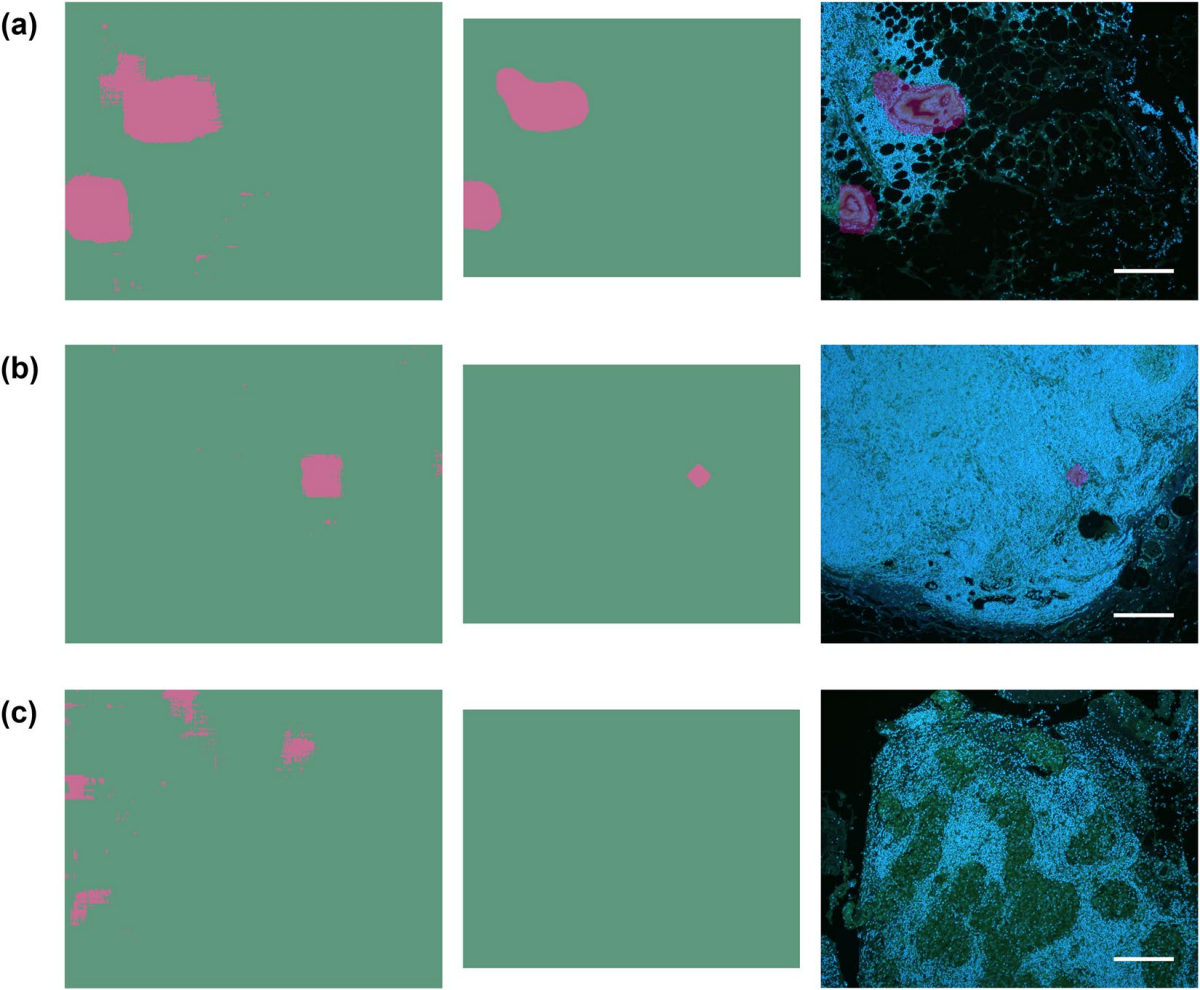
Comparison of mapping results with and without the majority decision image processing. All the cases correspond with the ones used in Fig. 5. The pink and green areas in the images of left and middle columns indicate the prediction pixels of metastasis-positive and -negative areas, respectively. Left column: maps of prediction results by the sliding-window technique. Middle column: the prediction maps after the majority decision image processing with the threshold of 70%. The sizes of the processed images were reduced by half the width of the 278 × 278 pixels window on four sides. Right column: overlays of the images in the left and middle columns. In the metastasis-positive cases (**a**,**b**), the metastatic lesions could be mapped more accurately. In metastasis-negative case (**c**), the sparse pixels misdiagnosed as metastatic were completely eliminated. Scale bars: 200 μm.

To validate the presented approach for a large-size image analysis, the undivided images were mapped by the sliding-window technique, and the majority decision model was subsequently applied. The representative results are shown in Fig. 6. With the majority decision model, the metastatic lesions could be mapped more accurately (Fig. 6a,b). In comparison, the sparse pixels misdiagnosed as metastatic were completely removed in non-metastatic LN (Fig. 6c).

## Discussion

DUV light is highly interactive with a biological tissue. This nature leads to intrinsic small penetration depth of DUV light into a tissue, allowing rapid superficial optical sectioning of a tissue block sample. The DUV surface excitation fluorescence microscopy enables wide-field imaging with rapid easy measurement. Moreover, fluorescent staining can be performed in short time. In fluorescence imaging, including with DUV excitation, there are a number of staining methods established^5–8,25^. Some of these staining methods allowed virtual H&E imaging like the presented Tb^3+^ and DAPI staining. However, among them, few can visualize nucleoli well. In addition, even if the nucleoli are visible, it takes time, and it is not easy to obtain reproducible data. Although there are studies showing that H&E-like images are acquired by scattering^26^, second harmonic generation^27^, multiphoton autofluorescence without using fluorescence^28^, they are not suitable for measuring wide-field area since they require scanning type of imaging. These methods typically require complicated and expensive instrumentation. On the other hand, the wide-field fluorescence imaging system used in this study is simpler and more inexpensive, which enhances commercial availability.

DNN-based analysis of medical images has recently been studied for automated diagnosis as well as support of clinicians’ diagnoses. In such studies, various kinds of images, including radiological images, magnifying endoscopic images, macroscopic optical images, and H&E images, have been used for detecting cancers in skin^29^, lungs^30,31^, gastrointestinal tracts^32–34^, and LNs^16–19^. In contrast, the present study employed fluorescence images representing molecular distributions for DNN-based analysis. Fluorescence imaging has potential strengths for DNN-based cancer detection; it can provide a high-contrast image by molecule-specific labeling. The labeling is not limited to subcellular structures but expanded to molecules, and hence molecular information as well as morphological information is obtained. Additionally, the multicolor technique provides rich information about a specimen with high image contrast. Furthermore, subcellular to cellular spatial resolutions allow sensitive detection of small neoplastic lesions. While similar strengths are also found in pathological stains such as H&E stain and immunohistochemical stain, some fluorescence stains are much faster than these stains. A combination of label-free imaging with multiphoton microscopy and DNN- based analysis for classification of hepatocellular carcinoma grading has been reported^35^. However, the histological information obtained by the label-free imaging is not enough compared to our fluorescent imaging approach. Some studies on cancer detection based on deep learning models using fluorescence imaging have already been reported^36,37^. However, the fluorescence *in situ* hybridization (FISH) and immunofluorescent staining techniques used in these papers require complicated processes and take time to obtain results, whereas the DUV excitation fluorescence imaging can be performed by a simple and rapid process. Therefore, we consider that DUV excitation fluorescence imaging is a more suitable technique for intraoperative rapid diagnosis. Furthermore, applications of fluorescence imaging are not limited to a microscope slide but include *in vivo* studies where a probe approved for use in the human body is used as well as an endogenous fluorescent compound that is targeted. *In vivo* fluorescence imaging of protoporphyrin IX, being useful for tumor detection in LNs^38,39^ as well as the brain and other tissues, is a potential combination of DNN-based analysis.

An important factor in diagnosis of LN metastasis is detecting epithelial cell components, which are not originally present in the LNs. Although cancer cells form various growth patterns as shown in Fig. 1, it is usually easy to distinguish cancer cells from lymphocytes which occupy the majority of LNs, because their cell bodies and nuclei are large. In the present study, however, histiocytes are an exceptional case to distinguish from metastatic lesions. In particular, the collection of histocytes can be confused with metastatic lesion, thus intracellular information is important in diagnosing cancer. In general, cancer cells show a higher nuclear-to-cytoplasmic ratio, nuclear pleomorphism, nucleomegaly, as well as larger and clearer nucleoli. In other words, the imaging of nuclei, nucleoli, and cytoplasm is minimally required for exact diagnosis of LN metastasis. It is epoch-making that good results were achieved with fluorescence images closely resembling H&E focused on nucleic acid molecule imaging, which is different from the conventional H&E staining mechanism. We expected that lymph follicles containing lymphoblasts having relatively abundant cytoplasm tended to be misdetected as metastatic lesions, but there was almost no misdiagnosis even without a majority decision model in this study.

The results of patch unit analysis indicated that the performance of DNN analysis with fluorescence images was comparable to that of DNN with H&E. We analyzed error images retrospectively. In the false-negative case (Suppl. Figure 4a), only a few cancer cells are in the patches. In Suppl. Figure 4b, connective tissues with gland-like structure may have been the cause of false-positive result. It is likely that the diagnostic accuracy will be further improved by incorporating additional images including such features in training dataset.

As a more practical model, we performed sliding-window technique with large-size images to aggregate the prediction of small patch units, and tried to construct a more improved model with additional data processing. In this study, the threshold to eliminate the pixels was derived from 100 images each for metastatic and non-metastatic lesions, and it is necessary to further increase the number of images. In addition, since this majority decision method removes metastatic pixels below the threshold, raising the threshold increases the incidence of false-negative result and lowering it increases that of false-positive result, as can be seen from Table 2. It would be ideally desirable to reduce both, but it is difficult to achieve with this method. We should consider the better ones, and accounting for the clinical setting, it is important to think about reducing the incidence of false-negative result, that is, misdiagnosis of cancer as non-cancer.

This study has a limitation in using fluorescence microscopy. Fluorescence observation inevitably causes photobleaching, which is not seen in the conventional H&E slides and bright-field microscopy. In this work, photobleaching was negligible due to the short exposure time, but it can lead to inhibition of the standardization of image quality. Optimization of the optical system should be continued, such as installing an automatic shutter to minimize the illumination on the samples, or placing a mask to occlude the excitation light so as not to illuminate the outside of the observation field.

In the future, we believe that this approach can be applied to analyze the images of other cancer types or surgical margins other than LN metastasis, because fluorescence imaging enables visualizing cell and tissue structures morphologically. In order to apply this approach to clinical practice, we require further investigation, e.g., on quick and reproducible sample preparation methods without overlooking metastatic lesions.

## Conclusion

Overall, this study demonstrates that the combination of DUV excitation fluorescence imaging and DNN analysis can be a useful tool for assisting pathologists in their classification of LN metastasis.

## Methods

### Clinical specimens

All clinical experiments were conducted with the approval of the Ethics Committees of Kyoto Prefectural University of Medicine (approval No. ERB-C-1038-1) as well as in accordance with guidelines from the committees and regional laws related to clinical research. The LNs used in this study were obtained from patients during gastric cancer surgery at University Hospital Kyoto Prefectural University of Medicine. Informed consent was obtained from all participants. The patients were diagnosed as metastasis-positive or -negative, based on postoperative pathological examination. None of the patients had received preoperative radiotherapy or chemotherapy. For developing a DNN model, 38 metastasis-positive and 18 metastasis-negative LNs of 18 patients were used. They were chosen to include all the common histologic types of gastric carcinoma^40^. For testing the sliding-window image processing method, 21 metastasis-positive and 6 metastasis-negative LNs of 13 patients were used. All the 83 LNs mentioned here were different LNs.

### Sample preparation and staining protocol

All the LNs used in this study were fixed with 10% formalin and embedded in solid paraffin blocks by clinical routine procedures. A portion of each paraffin-embedded block was used for clinical diagnosis, while portions of the remaining blocks were used for the DNN analysis. Each block was sliced into serial sections with approximate thicknesses of 4 μm. The sections were attached to glass slides individually. After deparaffinization, one section was treated with a conventional H&E staining protocol for each block. Each of the remaining consecutive sections was fluorescently stained for 3 min with a 100%-D_2_O HEPES-buffered solution containing TbCl_3_ (TBH03XB, Kojundo Chemical Laboratory) and DAPI (Dojindo Molecular Technologies) at the concentrations of 20 mM and 10 µg/ml, respectively^9^, and subsequently covered with a quartz coverslip.

### Fluorescence microscopy

We set the fluorescent sample on an inverted microscope (IX71, Olympus) equipped with an objective lens (UPLFLN 10 × , Olympus) to make the quartz coverslip face the objective. The sample was illuminated by a deep-UV beam (30 mW and 5 mm in power and diameter, respectively) emitting from an LED (M285L5, Thorlabs). Fluorescence emitting from the illuminated sample was collimated with the objective lens and imaged on a color CMOS camera (UI-3180CP-C-HQ Rev.2, OnSemi). From each sample, we acquired 1 to 45 camera images whose regions of measurements did not overlap each other, with a quick moving manual dual-axis stage having a precision of 1 µm.

### Image datasets for developing DNN models

Each of the fluorescence images acquired from 38 metastasis-positive and 18 metastasis-negative LNs was divided into the small patches composed of 278 × 278 pixels without overlapping of each other. Each patch was normalized by the maximum value over RGB channels. For the patches derived from the metastasis-positive LNs, two of the authors including an experienced pathologist examined whether the patches contained tumor cells, with referring to H&E images serially obtained from the same LNs, and subsequently selected the tumor-containing patches for metastasis-positive data. The number of patches we used as metastasis-positive and metastasis-negative data were 14,492 and 13,220, respectively.

From the same LNs, we prepared the small patches of H&E images similarly. For metastasis-negative LNs, three pathologists (excluding the pathologist providing the clinical diagnosis) confirmed that metastasis was not found. All the slides in two groups were converted to digital images by a digital slide scanner (Nanozoomer C9600-02; Hamamatsu Photonics) equipped with a 20 × objective lens (NA0.75). The digital images were converted to low-magnification ones so that the resultant image resolution (0.46 µm/pixel) was equivalent to that of the fluorescence images. We divided the low-magnification images into the small patches composed of 278 × 278 pixels without overlapping of each other. For metastasis-positive LNs, three experienced pathologists examined whether individual patches contained cancer cells or not, with consideration of several nearby patches, and subsequently we used only tumor-containing patches recognized by all three pathologists as metastasis-positive data. The numbers of patches we used as metastasis-positive and metastasis-negative data were 15,695 and 13,950, respectively.

### Training of DNN models

Three image recognition models of DNN were used, including VGG16, Inception v3, and Inception ResNet v2 provided by Keras. In each model, we downloaded the weights obtained by training with ImageNet’s image database and performed transfer learning. In the transfer learning of VGG16, only the full-connect (FC) layers were changed. The FC layers were composed of 32,768-dimensional (8 × 8 × 512) nodes generated by vectorization of each channel data, an intermediate layer of 256 nodes and a final layer of 2 nodes. The dropout rate^41^ of the middle layer was 0.5. In Inception v3 and Inception ResNet v2, output of the last inception module was processed by global average pooling^42^ and then joined to the final layer of 2 nodes. In the transfer learning of these three models, only the weights of FC layers were trained first, and then the weights of the whole model were trained. As an optimizer, stochastic gradient descent was used for all models. Each image patch was normalized before being input to the model. In all DNN models, training was performed with the training dataset and the weights were saved when the loss for the validation dataset was the smallest. In the evaluation using the test dataset, the weights were loaded.

### Method of creating mapping data

The mapping data was constructed by sliding a window of 278 × 278 pixels in 10 pixels steps, inputting the image of each sliding window into DNN, and sequentially obtaining the discrimination results. The term “window” as used here represents a rectangular area for cropping an image. In order to prevent disappearance of the metastasis-positive region present at the peripheral regions of the image in the later majority processing, mirroring of 139 pixels was performed on the top, bottom, left, and right of the original pathological image before performing the mapping processing. The size of the mapping data finally obtained by the above means was 259 × 204 pixels from the pathological image (2,592 × 2,048). When the step size of the sliding window was 35 pixels or 70 pixels, the size of the obtained mapping data was 74 × 58 and 37 × 29, respectively.

### Majority decision model

Images after majority processing are created from mapping data by Inception v3. Therefore, the deep learning model and the major decision model are separate processes. In the mapping data created with a sliding window of 10 pixels steps, a sufficiently small metastasis-positive area in the fluorescence pathology image is converted to a size of about 27 pixels square. Based on this size, a 27 × 27 pixels window was defined, and a new positive pixel was output when the ratio of the positive pixels included in the window exceeded the threshold. When the threshold is not exceeded, the pixel that is negative is output. The percentage of threshold was set every 10% from 40% to 90%. Similarly, in the case of mapping data created with a step size of 35 or 70 pixels, the window sizes for majority processing were 8 and 4 pixels square, respectively.

### PC and software used for calculation

For all calculations using artificial intelligence, a custom-made PC with a CPU (Core i7-8700) and a GPU (Geforce GTX 1080Ti, 11GB, NVIDIA) was used. The installed OS was Ubuntu 16.04 LTS. We used TensorFlow-gpu version 1.8.0 and Keras version 2.2.0 to build the DNNs. In order to obtain Grad-CAM data (Fig. 4), Keras-vis 0.4.1 was used.

## Supplementary information

Supplementary figures and tables
