# Supplementary material for: Deep-UV excitation fluorescence microscopy for detection of lymph node metastasis using deep neural network
Source: Sci Rep. 2019 Nov 15;9:16912. doi: 10.1038/s41598-019-53405-w (PMC6858352; doi:10.1038/s41598-019-53405-w)
Supplement: Supplementary file 1 — Supplementary figures and tables [file 41598_2019_53405_MOESM1_ESM.pdf]

Supporting Information

Deep-UV excitation fluorescence microscopy for detection of lymph node metastasis using deep neural network

Tatsuya Matsumoto[1,2,*], Hirohiko Niioka[3,*], Yasuaki Kumamoto[1], Junya Sato[4], Osamu Inamori[5], Ryuta Nakao[1], Yoshinori Harada[1], Eiichi Konishi[5], Eigo Otsuji[2], Hideo Tanaka[1], Jun Miyake[6], Tetsuro Takamatsu[1,7]

[1]Department of Pathology and Cell Regulation, Kyoto Prefectural University of Medicine, 465 Kajiicho, Kawaramachi-Hirokoji, Kamigyo-ku, Kyoto 6028566, Japan

[2] Division of Digestive Surgery, Department of Surgery, Kyoto Prefectural University of Medicine, 465 Kajiicho, Kawaramachi-Hirokoji, Kamigyo-ku, Kyoto 6028566, Japan

[3]Institute for Datability Science, Osaka University, 2-8 Yamadaoka, Suita, Osaka 5650871, Japan

[4] Faculty of Medicine, Osaka University, 2-2 Yamadaoka, Suita, Osaka 5650871, Japan

[5]Department of Surgical Pathology, Kyoto Prefectural University of Medicine, 465 Kajiicho, Kawaramachi-Hirokoji, Kamigyo-ku, Kyoto 6028566, Japan

[6]Global Center for Medical Engineering and Informatics, Osaka University, 1-3 Yamadaoka, Suita, Osaka 5650871, Japan

[7]Department of Medical Photonics, Kyoto Prefectural University of Medicine, 465 Kajiicho, Kawaramachi-Hirokoji, Kamigyo-ku, Kyoto 6028566, Japan

*These authors equally contributed to this work.

Table of Contents

Supplementary figures

Figure 1. Workflow of the proposed DNN analysis for detection of lymph node metastasis.

Figure 2. Accurate classification of fluorescence images.

Figure 3. DNN structure based on Inception v3.

Figure 4. Example of cases misdiagnosed by the algorithm.

Figure 5. t-SNE visualization of the last hidden layer representations in the DNN for H&E images.

Supplementary tables

Table 1. Data distribution.

Table 2. True prediction rates for large-size images processed with the majority decision model having various thresholds (the sliding-window step sizes of 35 and 70 pixels).

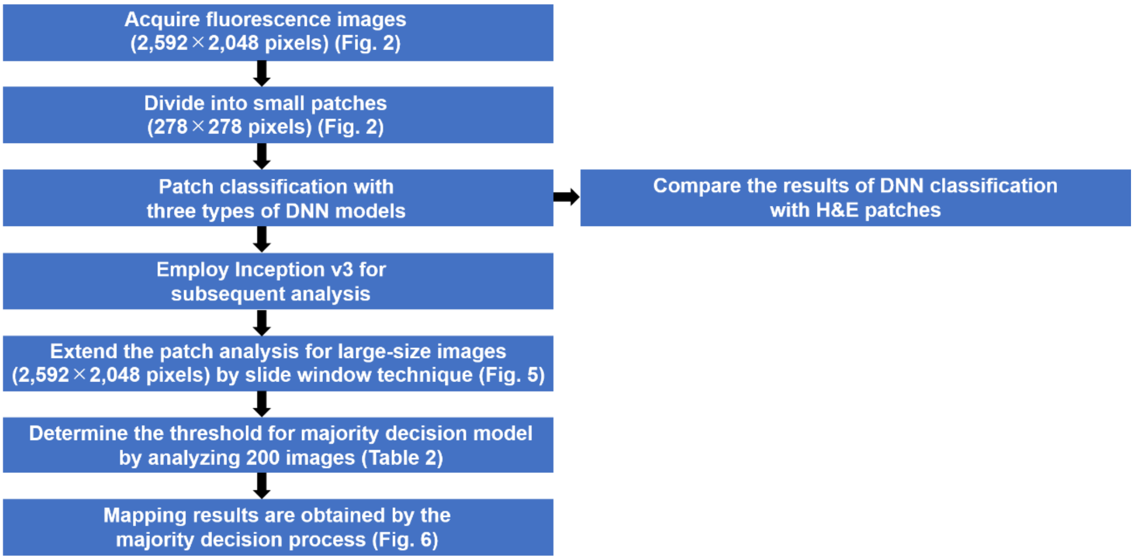

Supplementary Figure 1. Workflow of the proposed DNN analysis for detection of lymph node metastasis. First, fluorescence images were divided into patches, and a DNN model for classifying metastasis-positive/-negative with high accuracy at the patch level was constructed. Next, the majority decision model for detecting metastasis in large-size images was established.

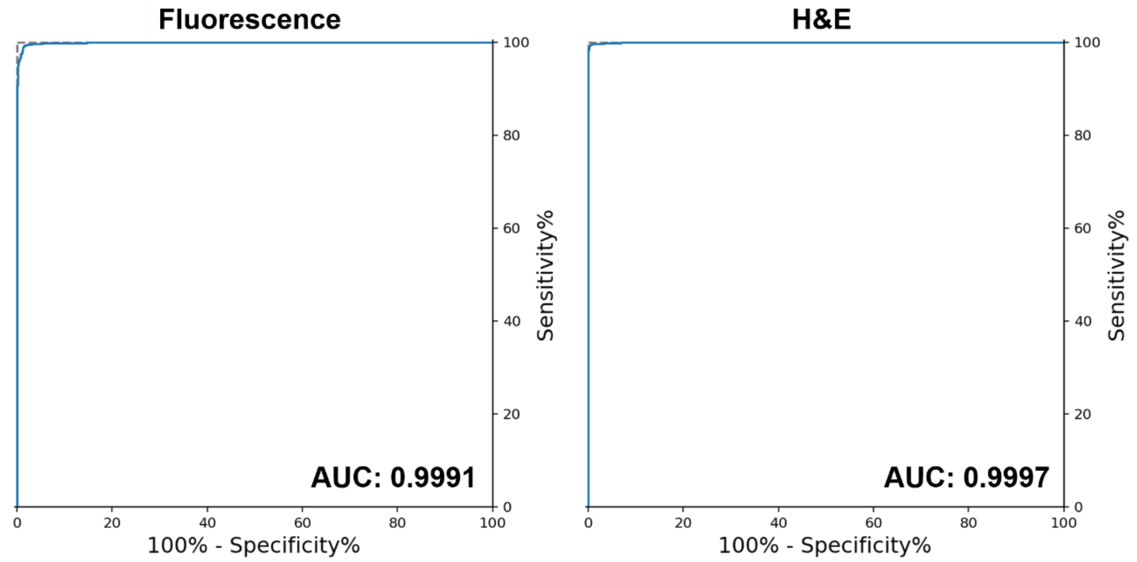

Supplementary Figure 2. Accurate classification of fluorescence images. The AUC of fluorescence images was 0.999, and comparable to that of H&E.

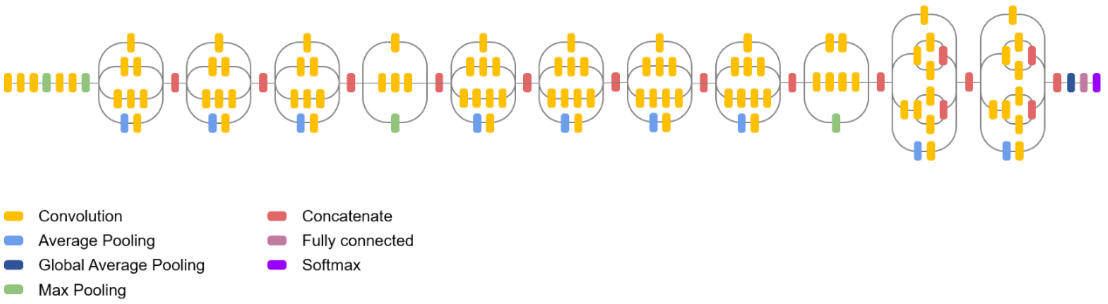

Supplementary Figure 3. DNN structure based on Inception v3. We used a model of the Inception v3 structure provided by the Keras framework. This model was modified as follows, the fully connected layer and the softmax layer were connected after the last global average pooling layer, and the final layer was converted from the original 1,000 nodes to 2 nodes. Image data is input from the left of the figure, and after passing through

layers such as convolution layers, the probability of LN metastasis and normal LN is output after passing through the last softmax layer. The diagnostic result was output with 0.5 as the threshold.

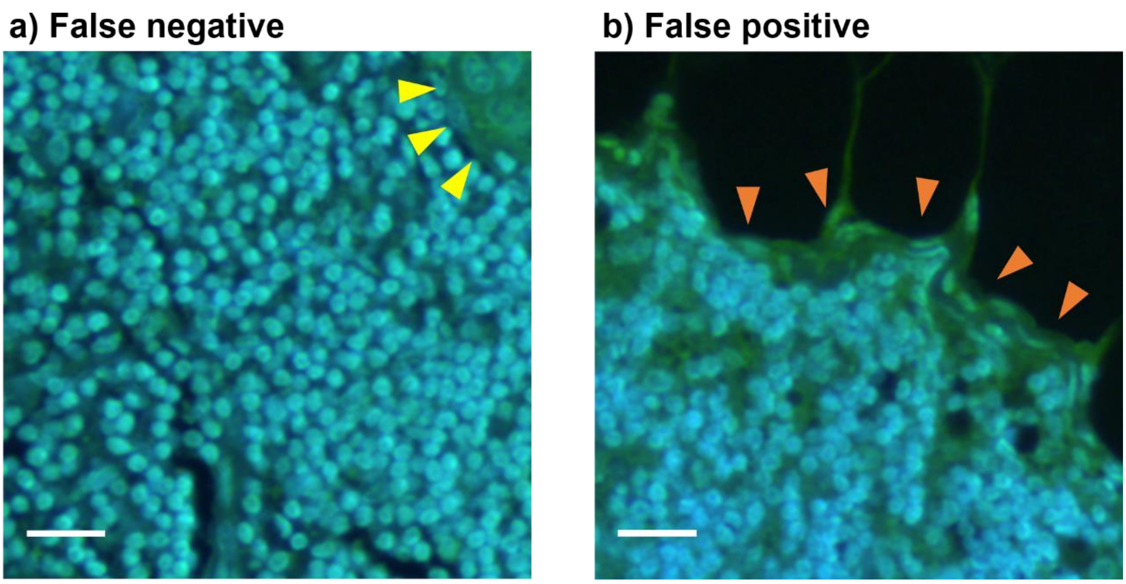

Supplementary Figure 4. Example of cases misdiagnosed by the algorithm. (**a**) A few cancer cells (yellow arrowheads) are located at the upper right corner in the image. (**b**) Connective tissues (orange arrowheads) surrounding LNs are similar to glandular structure. Scale bars, 20 μm

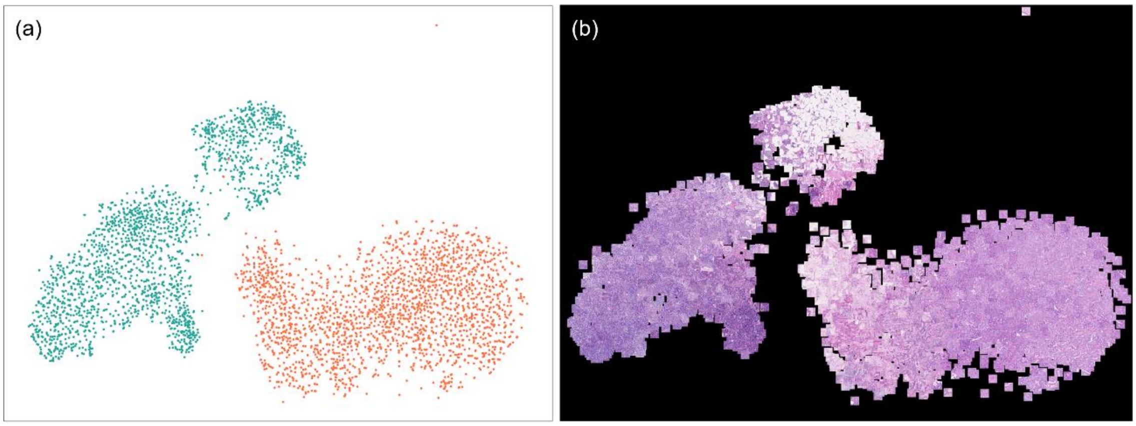

Supplementary Figure 5. t-SNE visualization of the last hidden layer representations in the DNN for H&E images. (**a**) Scatterplots generated by the deep learning network for classification of metastasis positive and negative via the t-SNE algorithm. The orange and green plots indicate the prediction of metastasis-positive and -negative, respectively. (**b**) Patches corresponding to the points are embedded. N=4,330 images (test data) of 38 metastasis-positive and 18 metastasis-negative LNs from 20 patients.

Supplementary Table 1. Data distribution.

	Training	Validation	Test
Fluorescence metastasis (+)	10507	1991	1994
Fluorescence metastasis (-)	9931	1455	1834
H&E metastasis (+)	11169	2230	2296
H&E metastasis (-)	10116	1800	2034

n=38 metastasis (+) and 18 metastasis(-) LNs from 20 patients

Supplementary Table 2. True prediction rates for large-size images processed with the majority decision model having various thresholds (the sliding-window step sizes of 35 and 70 pixels).

	Threshold	True prediciton rates	
		Metastasis-positive	Metastasis-negative
35 pixels slide	40%	98/100	82/100
	50%	97/100	86/100
	60%	96/100	94/100
	70%	95/100	95/100
	80%	93/100	98/100
	90%	87/100	100/100
70 pixels slide	40%	98/100	77/100
	50%	97/100	88/100
	60%	97/100	93/100
	70%	95/100	96/100
	80%	92/100	96/100
	90%	90/100	98/100
